# Healthcare Provider Discrimination toward Pregnant Women with Rifampin-Resistant Tuberculosis

**DOI:** 10.3201/eid2503.181571

**Published:** 2019-03

**Authors:** Marian Loveday, Sindisiwe Hlangu, Jennifer Furin

**Affiliations:** South African Medical Research Council, Cape Town, South Africa (M. Loveday, S. Hlangu);; Harvard Medical School, Boston, Massachusetts, USA (J. Furin)

**Keywords:** pregnancy, healthcare, discrimination, tuberculosis and other mycobacteria, TB, rifampin-resistant, antimicrobial resistance, South Africa, bacteria

## Abstract

Little is known about the treatment experiences of pregnant women with rifampin-resistant tuberculosis. We conducted qualitative interviews with 10 women who had this condition; 9 reported facing discrimination from healthcare providers. Our findings underscore an urgent need to ensure a human-rights–based, patient-centered approach for women with rifampin-resistant tuberculosis who are pregnant.

We report discrimination by healthcare providers toward pregnant women with rifampin-resistant tuberculosis (TB) in KwaZulu-Natal Province, South Africa. Pregnant women are at increased risk for all forms of tuberculosis (*1*). Experience in caring for pregnant women with rifampin-resistant TB is limited (*2*–*4*), and little is known about their experiences during treatment (*5*).

In 2016, a total of 3,280 patients at the King Dinuzulu Hospital Complex in KwaZulu-Natal Province were started on treatment for rifampin-resistant TB; 47% of these patients were women, many in their childbearing years (*6*). During 2016–2017, we conducted qualitative interviews with 10 women as part of an observational study on bedaquiline use during pregnancy, with the goal of describing treatment challenges. 

All 10 of the women were postpartum and ranged in age from 22 to 37 years. The mean number of pregnancies was 3 (range 2–5). Two women were on rifampin-resistant TB treatment when they became pregnant, 7 women had rifampin-resistant TB diagnosed during pregnancy, and 1 had rifampin-resistant TB diagnosed and found out she was pregnant at the same time. Nine of the 10 women reported experiencing discrimination while accessing healthcare, either from the antenatal care providers (9 women), the TB care providers (4 women), or both (4 women). (Rifampin-resistant TB and antenatal care services are not integrated in this setting.)

The reported discrimination faced by the women while accessing antenatal care and during delivery was largely based on a fear of resistant TB transmission. Even when the women were no longer infectious, they were forced to wear masks, the nurses caring for them began wearing gloves, and they were isolated during the delivery and in the postpartum period. Three women reported that they were not evaluated after delivery and that they were left alone without food for themselves or milk for their newborns. Women also reported that they felt pressured into considering pregnancy termination by their antenatal care providers, who sometimes referred to their unborn children as “that thing”; however, none of the women interviewed terminated their pregnancy.

Most of the women interviewed also reported discrimination from rifampin-resistant TB care providers. Some providers told the women they were “foolish” for becoming pregnant during treatment. Several of the women also reported that some care providers ridiculed them for continuing with their pregnancies, stating “We don’t know what [you] will give birth to.” Some women were offered incomplete treatment regimens (e.g., regimens that were missing >1 drugs for part of the treatment period, including ethionamide, injectable agents, or bedaquiline). Some women experienced a delay in the start of rifampin-resistant TB treatment or were started on first-line therapy even though they had a confirmed diagnosis of resistant TB.

Although the number of women in this study is small and their experiences might not be representative of all pregnant women, the common experience of reported discrimination is cause for concern. Proper infection control practices are needed to reduce nosocomial and community transmission of rifampin-resistant TB, but most of these women reported that the discrimination occurred even after they were unlikely to be infectious (*7*). 

Some of the discriminatory acts might have placed these women and their newborns at increased risk for poor health outcomes, colored their perceptions of the healthcare systems, and adversely affected future healthcare-seeking behavior for themselves and their children. Additional exploration of this phenomenon is needed along with practical solutions to ensure pregnant women with rifampin-resistant TB receive the best possible care during this challenging period of their lives.

## References

[1] Mathad JS, Gupta A. Tuberculosis in pregnant and postpartum women: epidemiology, management, and research gaps. Clin Infect Dis. 2012;55:1532–49. 10.1093/cid/cis732 22942202 PMC3491857

[2] Palacios E, Dallman R, Muñoz M, Hurtado R, Chalco K, Guerra D, et al. Drug-resistant tuberculosis and pregnancy: treatment outcomes of 38 cases in Lima, Peru. Clin Infect Dis. 2009;48:1413–9. 10.1086/598191 19361302 PMC4824949

[3] Khan M, Pillay T, Moodley J, Ramjee A, Padayatchi N. Pregnancies complicated by multidrug-resistant tuberculosis and HIV co-infection in Durban, South Africa. Int J Tuberc Lung Dis. 2007;11:706–8. 17519106

[4] Tabarsi P, Moradi A, Baghaei P, Marjani M, Shamaei M, Mansouri N, et al. Standardised second-line treatment of multidrug-resistant tuberculosis during pregnancy. Int J Tuberc Lung Dis. 2011;15:547–50. 10.5588/ijtld.10.0140 21396217

[5] Zumla A, Bates M, Mwaba P. The neglected global burden of tuberculosis in pregnancy. Lancet Glob Health. 2014;2:e675–6. 10.1016/S2214-109X(14)70338-9 25433613

[6] Pillay T, Khan M, Moodley J, Adhikari M, Padayatchi N, Naicker V, et al. The increasing burden of tuberculosis in pregnant women, newborns and infants under 6 months of age in Durban, KwaZulu-Natal. S Afr Med J. 2001;91:983–7. 11847922

[7] Dharmadhikari AS, Mphahlele M, Venter K, Stoltz A, Mathebula R, Masotla T, et al. Rapid impact of effective treatment on transmission of multidrug-resistant tuberculosis. Int J Tuberc Lung Dis. 2014;18:1019–25. 10.5588/ijtld.13.0834 25189547 PMC4692272

